# A sandwich structure composite wound dressing with firmly anchored silver nanoparticles for severe burn wound healing in a porcine model

**DOI:** 10.1093/rb/rbab037

**Published:** 2021-08-03

**Authors:** Jianmin Yang, Yufeng Huang, Jiajia Dai, Xianai Shi, Yunquan Zheng

**Affiliations:** 1 Department of Biomedical Engineering, College of Biological Science and Engineering, Fuzhou University, No. 2 Xueyuan Road, Fuzhou 350108, China; 2 Fujian Key Lab of Medical Instrument and Biopharmaceutical Technology, Fuzhou University, No. 2 Xueyuan Road, Fuzhou, 350108, China; 3 Institute of Pharmaceutical Biotechnology and Engineering, College of Chemistry, Fuzhou University, No. 2 Xueyuan Road, Fuzhou 350108, China

**Keywords:** burn injury, wound dressing, silver nanoparticle, antimicrobial activity

## Abstract

Wounds may remain open for a few weeks in severe burns, which provide an entry point for pathogens and microorganisms invading. Thus, wound dressings with long-term antimicrobial activity are crucial for severe burn wound healing. Here, a sandwich structure composite wound dressing anchored with silver nanoparticles (AgNPs) was developed for severe burn wound healing. AgNPs were *in situ* synthesized on the fibers of chitosan nonwoven fabric (CSNWF) as the interlayer of wound dressing for sustained release of silver ion. The firmly anchored AgNPs could prevent its entry into the body, thereby eliminating the toxicity of nanomaterials. The outer layer was a polyurethane membrane, which has a nanoporous structure that could maintain free transmission of water vapor. Chitosan/collagen sponge was selected as the inner layer because of its excellent biocompatibility and biodegradability. The presence of AgNPs in the CSNWF was fully characterized, and the high antibacterial activity of CSNWF/AgNPs was confirmed by against *Escherichia coli*, *Pseudomonas aeruginos*a and *Staphylococcus aureus*. The superior wound healing effect on deep dermal burns of presented composite wound dressing was demonstrated in a porcine model. Our finding suggested that the prepared AgNPs doped sandwich structure composite wound dressing has great potential application in severe wound care.

## Introduction

Burn injuries, caused by thermal, electricity, friction, chemical agents, radiation, etc., are a common global health problem and present an enormous medical and financial burden. Burn-initiated pathophysiological events can raise the risk of disfigurement, functional impairment and serious complications by increasing vascular permeability and fibrinolysis, changing in extravasation of plasma proteins and platelet aggregation [1]. According to the World Health Organization, ∼180 000 deaths worldwide annually are related to burn injuries, and 11 million people worldwide were burned severely enough to require medical treatment [2]. Generally, epidermal burns and superficial partial-thickness burns tend to heal within 2 or 3 weeks with moderate treatment, whereas severe burns, including deep dermal burns and full-thickness burns, often require an extended period for healing [3, 4]. In deep dermal burns, wounds may remain open for a few weeks, which provide an entry point for pathogens and microorganisms invading [5]. Consequently, biofilms formed, thereby delaying wound closure by triggering systemic inflammatory and immunological responses [6]. Apart from the hypertrophic or keloid scars formation, biofilms can exacerbate wound infection and lead to life-threatening complications, such as sepsis, respiratory failure and multi-organ failure [7–9]. Therefore, simultaneous inhibition of biofilm formation and promotion of wound healing is paramount to deep dermal burns treatment.

Antimicrobial wound dressings are developed to prevent wound infection by minimizing bacterial colonization and provide proper coverage for the injured area to aid reepithelialization. Various antimicrobial dressings containing antibiotics or antimicrobial agents have been widely applied for severe and chronic wound healing [10–14]. Undoubtedly, antibiotics have played a critical role in bacterial inhibition in the last decades [15]. However, the emergence of drug-resistant bacteria, like methicillin-resistant *Staphylococcus aureus* and vancomycin-resistant *Enterococci*, has become a major global public health challenge [16–18]. Although alternative antimicrobial agents such as silver ion (Ag^+^) [19], cadexomer iodine [20], curcumin [21] and antimicrobial peptides [22, 23] have significant antimicrobial activity without compromised wound healing, the burst release of agents at the wound site could enhance the adverse effect of agents and result in organs damage [24–26]. Besides, frequent dressing changes were required to maintain the antimicrobial effect for wound bed, which increases the nursing costs and patient’s suffering, as well as delays healing by traumatizes the newly formed epithelial surface.

Recently, wound dressings with long-term antimicrobial activity have been developed by the incorporation of nanoscale antimicrobial materials into tissue-engineered scaffolds [27–29]. Among them, silver nanoparticles (AgNPs) are promising antimicrobial nanomaterials to have broad-spectrum antimicrobial activity and anti-inflammatory properties [30–33]. However, compromised antimicrobial activity usually occurs because of the aggregation and precipitation of AgNPs, which leads to a dramatically reduced exposed surface area [34]. Moreover, the toxicity of AgNPs should not be ignored once it enters the human body, which has significant damages on multi-visceral organs like spleen, liver and kidney [35]. Hence, it is essential to improve the stability of AgNPs in wound dressings for controlled antimicrobial activity and minimal toxicity.

The majority of studies on wound dressings were the selection of small animals such as rats and rabbits as clinically relevant wound models to assess wound healing ability. Although dramatic healing performance on these small animal wound models was demonstrated, the effectiveness of presented dressings on humans should be cautioned. For instance, rats are irrelevant to clinical wound healing because they have loose skin and show high resistance to infection. In addition, wound healing in rats is mainly dependent on wound contracture rather than scar tissue formation, whereas the opposite process occurs in humans because the humans’ skin has less elasticity [36, 37]. It should be noted that the concordance of wound healing mechanism between humans and small mammal cutaneous was only 53%, while humans and pigs were as high as 78% [38]. Therefore, evaluation of wound healing in a porcine model can provide a more effective prediction of clinical treatment quality, because of the high level of similarity of pigs and humans in anatomy and physiological structure, innate and adaptive immunity and metabolic characteristics [39].

**Scheme 1.**
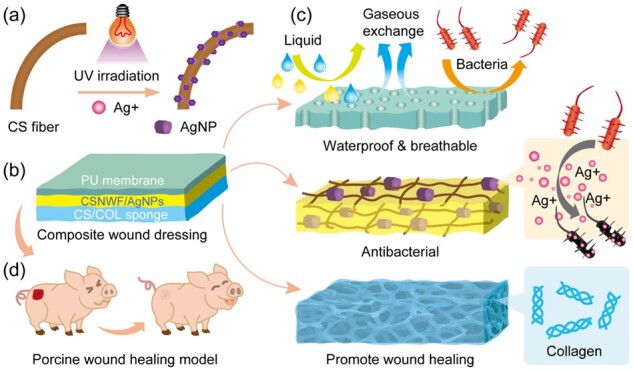
Schematic diagram of AgNPs anchored sandwich structure composite wound dressing PU-CSNWF/AgNPs-CS/COL. (**a**) *In situ* synthesis of AgNPs on the fiber of CSNWF. (**b**) The sandwich structure of composite wound dressing and (**c**) function of each layer. (**d**) Severe burn wound healing model in porcine

In this work, an AgNPs anchored sandwich structure composite wound dressing with high antimicrobial activity was prepared for severe burn wound healing in a porcine model. As illustrated in Scheme 1, the outer layer of wound dressing was a polyurethane (PU) membrane. The nanoporous structure and superior stretchability of the PU membrane could prevent external bacteria, oil, water and dust invasion and contamination, while maintaining free transmission of water vapor and providing sufficient mechanical strength [40]. Chitosan (CS) and collagen (COL) were selected for preparation of a sponge as the inner layer because of their excellent biocompatibility and biodegradability. The intrinsic antibacterial activity of CS can decrease the risk of infection, while the coagulation activity of CS and cytocompatibility of COL could accelerate the wound healing process [41–43]. Moreover, the porous CS/COL sponge can remove exudates and maintain a moist wound milieu. An AgNPs anchored chitosan nonwoven fabric (CSNWF) as an interlayer for sustained release of Ag^+^. The AgNPs were *in situ* synthesized on the fibers of CSNWF and showed strong adhesion capability, which can prevent the detachment and aggregation of AgNPs. Meanwhile, the CSNWF/AgNPs as interlayer could prevent the AgNPs direct contact with wound tissue and thereby avoiding the toxicity of AgNPs to the human body. The wound healing effect on deep dermal burns of presented composite wound dressing was validated in a porcine model.

## Materials and methods

### Reagents and materials

CSNWF (120 g/m^2^) was purchased from Hismer Bio-Technology Co., Ltd. (Shandong, China). CS (M_w_ = 5 and 50 kDa, degree of deacetylation = 91.6%) was purchased from Nantong Xingcheng Biological Industry Limited Co. (Jiangsu, China). COL (M_w_ = 2 and 100 kDa) was purchased from Haishen Bio-Technology Co., Ltd. (Fujian, China). Medical PU membrane with glue on one side was purchased from Sanrui Medical Device Co., Ltd (Jiangsu, China). Silver sulfadiazine (SSD) cream was obtained from Kunming Huarun Shenghuo Pharmaceutical Co., Ltd. (Yunnan, China). Ketamine, factor VIII kit, vascular endothelial growth factor assay (VEGF) kit, nitric oxide (NO) kit and endothelin kit were provided by the Union Hospital of Fujian Medical University (China). AgNO_3_, HNO_3_ and ammonia were obtained from Sinopharm Chemical Reagent Co., Ltd. (Shanghai, China).

The human skin fibroblast (HSF) cell line was obtained from Dingguo Biotechnology Co., Ltd. (Beijing, China). Cellstain live/dead double staining kit was purchased from BestBio Co., Ltd. (Shanghai, China). The WST-1 reagent was obtained from Roche Molecular Biochemicals (Mannheim, Germany). *Escherichia coli* (ATCC-8739), *S.aureus* (ATCC-14458) and *Pseudomonasaeruginosa* (CMCC B 10104) were purchased from Luwei microbial Sci. & Tech Co., Ltd. (Shanghai, China). Fetal bovine serum (FBS) was purchased from Gibco Laboratories (Grand Island, NY, USA). Dulbecco's modified Eagle’s medium (DMEM), L-glutamine, penicillin, streptomycin, trypsin/EDTA and dimethyl sulfoxide were purchased from Sigma-Aldrich (St Louis, MO, USA).

### Preparation of composite wound dressing

The process of composite wound dressing preparation was illustrated in Supplementary Fig. S1. It mainly includes the *in situ* growth of AgNPs on the CSNWF, CS/COL sponge preparation and fabrication. 

#### *In situ* synthesis of AgNPs on the CSNWF

UV-assisted synthesis method was employed to *in situ* grow AgNPs on the commercial CSNWF. First, CSNWF with a size of 10 cm × 10 cm was quickly washed (1 min) with 5 mM HNO_3_, followed by thorough rinsing with water several times. Afterward, the pretreated CSNWF was soaked into 50 ml of AgNO_3_ aqueous solution for 1 h with the Ag^+^ concentration of 5, 10 and 20 mM, respectively. Subsequently, the CSNWF was exposed to UV irradiation (365 nm, 10 W). After UV irradiation, the AgNPs loaded CSNWF was washed with water and then dried under vacuum at room temperature.

#### CS/COL mixture preparation

Two different molecular weights of CS (5 and 50 kDa) and COL (2 and 100 kDa) were used to prepare the CS/COL sponges without any crosslinking reaction. The mass fraction of high molecular weight CS and COL accounted for 90%. Here, the high molecular weight polymers can enhance the mechanical properties and stability of CS/COL sponges. The incorporated low molecular weight polymers were more beneficial to release and absorption, thereby promoting wound healing. A mount of 0.9 g of CS (M_w_ = 50 kDa) was dissolved in 100 ml of 0.5% acetic acid solution with constant stirring. Subsequently, 0.1 g CS (M_w_ = 5 kDa), 0.9 g COL (M_w_ = 100 kDa) and 0.1 g COL (M_w_ = 2 kDa) were dissolved into the mixture. After thoroughly mixing, the CS/COL mixture was placed overnight under vacuum to eliminate any air bubbles.

#### Sandwich structure composite wound dressing fabrication

One side of CSNWF/AgNPs was sprayed with 10 ml of water and then frozen at −80°C for 0.5 h. Afterward, the homogeneous CS/COL mixture (10 ml) was immediately sprayed on the side of CSNWF/AgNPs which was sprayed with water and then frozen 1 h under −80°C. After lyophilization, the CS/COL sponge combined CSNWF/AgNPs matrix was obtained. Finally, the sandwich structure composite wound dressing was prepared by adhering to the CSNWF/AgNPs-CS/COL matrix to a medical PU membrane.

### Antimicrobial activity evaluation

#### Inhibition zone method

The CSNWF/AgNPs was cut into 1.0 cm diameter disks and sterilized by 70% aqueous ethanol solution for 40 min, followed by thorough washing with PBS. After spreading 100 μl of 1 × 10^8^ CFU/ml bacterial suspension (*E.coli*, *P.aeruginosa* and *S.aureus*) on LB agar plate, the sterile CSNWF/AgNPs disks were placed onto the surface of the agar. After culture 24 h at 37°C, the diameter of the inhibition zone was recorded.

#### Shake flask method

The tested CSNWF/AgNPs was cut into 1.0 cm × 1.0 cm pieces and sterilized as the method mentioned above. After CSNWF/AgNPs piece immersed into 100 ml of PBS, 5 ml of 1 × 10^5^ CFU/ml bacterial suspension (*E.coli*, *P.aeruginosa* and *S.aureus*) was added and mixed thoroughly. Subsequently, the mixture was placed in a shaker under 25°C with constant shaking at 150 rpm. After 24 h incubation, 1 ml of supernatant was taken to determine the number of bacteria. The supernatant was serially diluted by PBS and 100 μl of the solution was placed on agar plates, on which the grown colonies were counted for analysis. The mixture of PBS and bacterial suspension without any AgNPs/CSNWF as the control group. 

### *In vitro* cytocompatibility and wound healing study

The *in vitro* cytocompatibility and wound healing of prepared wound dressing were evaluated by culture the HSF cells in the leaching solution of the composite wound dressing according to our previous study [44]. The leaching solution was obtained by incubation of sterilized wound dressing (5.0 cm × 5.0 cm) in 50 ml of serum-free culture medium for 24 h under 37°C. The serum-free culture medium was DMEM supplemented with 100 units/ml penicillin and 100 mg/ml streptomycin.

#### Cytocompatibility study

A volume of 100 μl HSF cells suspension in culture medium was seeded into a 96-well plate at a density of 1 × 10^5^ cells/mL, and then cultured at 37°C under a humidified atmosphere containing 5% CO_2_. After being cultured 12 h, the culture medium was replaced by 100 μl of the leaching solution with 10% of FBS. After incubated at different times, the leaching solution was decanted, and cells were washed with PBS. The viability and proliferation of HSF cells were measured by the live/dead staining and WST-1 assay. As for live/dead staining, 100 μl of live/dead staining stock solution was added to each well and then incubated for 15 min at 4°C. Afterward, the fluorescent images of cells were viewed using a fluorescence microscope (TE2000-S, Nikon, Japan). For WST-1 assay, 100 µl of culture medium includes 10 μl of WST-1 reagent was added to each well. After incubation for 4 h, the optical intensity in each well was measured using a microplate reader (SH1000, Corona, Japan) at 450 nm.

#### Scratch wound assay

The *in vitro* wound healing ability of prepared wound dressing was analyzed by scratch test. HSF cells were seeded into a six-well plate at a density of 1 × 10^5^ cells per well and cultured with culture medium for 24 h at 37°C under a humidified atmosphere containing 5% CO_2_. After the scratch was made, the culture medium was removed, and the cells were washed with PBS. Subsequently, the PBS was decanted, and 2 ml of leaching solution was added into each well. Microscopy images were taken using the bright-field channel every 6 h and the area of the void between cells was measured using ImageJ software.

### *In vivo* porcine burn models

#### Animals

All animals were treated humanely throughout all experiments. All protocols were reviewed and approved by the Institutional Ethics Committee for Animal Research of Fujian Medical University. A number of eight ∼12-week-old male pigs (∼20 kg) provided by the Laboratory Animal Center of Fujian Medical University were used in this study. All animals were delivered to the animal house and housed in an individual cage for 5 days with a 12-h light/dark cycle for acclimatization. The room temperature was maintained at 25°C with a humidifying of 50%. Animals were given free access to water and fed a standard grower feed pellet diet throughout the experimental periods.

#### Deep second-degree burn model and experimental protocol

Pigs fasted overnight on days before surgical procedures. The burn model was performed under general anesthesia with Ketamine *via* intramuscular injection. The dorsal surface of the pig was shaved and sterilized with 0.1% benzalkonium bromide solution. Afterward, serve square scald wounds were created using a scald device by placing it on the dorsal skin of pigs for 25 s at 100°C. Burns with a size of 2.0 cm × 2.0 cm (wound interval was 3–4 cm) were located on the dorsal of the pig, with ten burns on each side, giving a total of 20 burns per pig (Supplementary Fig. S2a).

After burns were numbered, the wounds were randomly covered with medical cotton gauze, gauze-SSD, CSNWF, CSNWF/AgNPs, CS/COL sponge and CSNWF/AgNPs-CS/COL sponge, and then fixed by medical PU membrane (Supplementary Fig. S2b). Custom made garments were fitted further to protect the burn area (Supplementary Fig. S2c). Wound dressing changes were performed on Days 3, 7, 10, 14, 17, 21, 24 and 28 post-burn, biopsies were taken on Days 7, 14, 21 and 28. At dressing changes and biopsy collection, burns were rinsed with saline and then cleaned by removing exudate and dried eschar with sterile swabs soaked in 0.1% chlorhexidine gluconate solution. Animals underwent the same general anesthetic regime at each dressing change and biopsy collection time point. At the endpoint of each experiment, animals were euthanized with sodium pentobarbitone.

#### Wound closure measurement

On Days 7, 14, 21 and 28 post-burn, the wounds were drawn out by transparent graph paper. Afterward, the drawing of wounds was scanned, and the wound surface areas were measured using Adobe Photoshop and Osiris software. Wound healing rate over time was expressed as the percentage of the initial wound areas.

#### Histology staining

The collected skin tissue on burns was rinsed with PBS and then fixed in 4% paraformaldehyde. After dehydrated in a graded series of ethanol and dimethyl benzene, the skin was embedded into paraffin blocks and cut into sections with a thickness of 4 μm. The skin sections were stained routinely with hematoxylin and eosin (H&E), and then viewed and photographed with an optical microscope (Nikon Eclipse CI) equipped with an image-forming system (Nikon DS-U3).

#### Immunohistochemical evaluation of the microvascular density

Immunohistochemical staining of paraffinic skin sections was performed with factor VIII according to the manufacturer's protocol. Subsequently, skin sections were examined by a microscope, and the microvascular sections were counted. The microvascular density was expressed as the average of 10 fields examined.

#### Endothelin, NO and VEGF assay

After removing the dried eschar and bloodstain, 100 mg of collected skin tissue was homogenized in 1 ml sterile saline. For endothelin assay, the specimens were centrifuged at 3000 rpm for 15 min under 4°C, and the supernatant was stored at −20°C. For NO and VEGF assay, the specimens were centrifuged at 10 000 rpm for 15 min under 4°C, and the supernatant was stored at −20°C.

### Statistical analysis

All the analytical determinations were carried out at least in triplicate, and the results were reported as means ± standard deviation. All statistical computations were performed using the SPSS 19.0 software. Statistical comparisons were determined by using one-way ANOVA statistical analysis. Value of * (*P* < 0.05), ** (*P* < 0.01) and *** (*P* < 0.001) were considered to be statistically significant.

## Results and discussion

### *In situ* growth of AgNPs on the fibers of CSNWF

**Figure 1.**
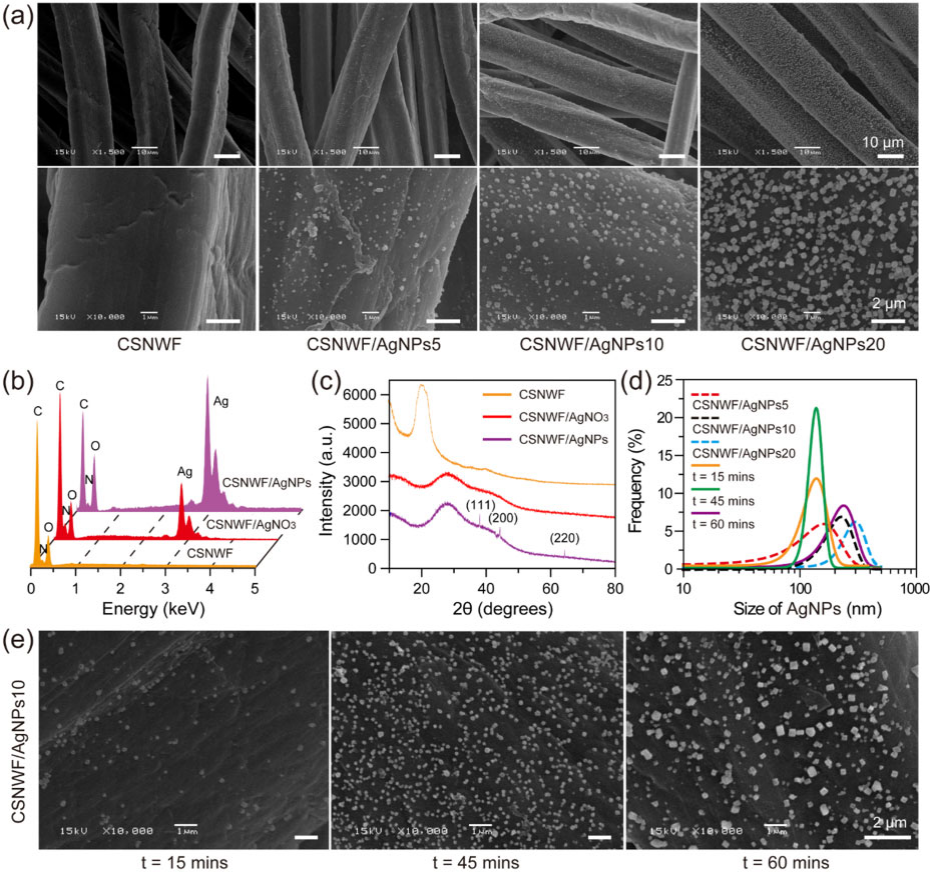
(**a**) Scanning electron microscope (SEM) images of original and AgNPs anchored fibers of CSNWF. Groups of CSNWF/AgNPs5, CSNWF/AgNPs10 and CSNWF/AgNPs20 represented the CSNWF were treated by the AgNO_3_ solution at the concentration of 5, 10 and 20 mM, respectively. The UV irradiation time was 30 min. (**b**) EDS and (**c**) XRD characterization. (**d**) The size distribution of AgNPs in different conditions. (**e**) SEM images of the CSNWF/AgNPs10 group with different duration of UV irradiation

AgNPs tend to aggregate and/or coalescence due to the high surface energy, high reactivity and strong cohesiveness of nanoparticles [45]. Hence, the aggregated AgNPs leading to the decreased exposed surface area, and thereby reducing antibacterial activity. Here, a UV-induced reduction approach was employed to *in situ* growth of AgNPs on the CSNWF to prevent AgNPs aggregation and accumulation. Commercial available CSNWF consists of uniform microfibers with a diameter of 18.3 ± 2.5 μm (Supplementary Fig. S3). The surface of the original fibers was smooth and clean, while numerable nanoparticles were found after the procedures of AgNO_3_ impregnation and UV irradiation (Fig. 1a). It should be noted that few and amorphous Ag particles were obtained when the CSNWF without HNO_3_ pretreatment (Supplementary Fig. S4). We inferred that the HNO_3_ could remove any impurities and residual CS fragments on the surface of fibers, and thus prompt AgNPs formation and adhesion. Energy dispersive spectrometer (EDS) characterization results indicated that the Ag presented on the fibers of CSNWF after AgNO_3_ impregnation and UV irradiation treatment (Fig. 1b). Moreover, X-ray diffraction (XRD) was employed to confirm the incorporation of AgNPs into CSNWF. As shown in Fig. 1c, the distinct diffraction peaks at 38, 45 and 64 were observed in the CSNWF/AgNPs, which corresponded to the (111), (200) and (220) crystal planes of AgNPs. In contrast, no obvious diffraction peaks could be observed in the CSNWF and CSNWF/AgNO_3_. The results revealed the presence of AgNPs after UV irradiation.

The formation of AgNPs on the fibers of CSNWF was thought to start with the interaction of the substrate with Ag^+^. The AgNO_3_ solution was absorbed by the CSNWF due to capillary forces and facilitated by CS’s hydrophilic hydroxyl and amine groups. Ag^+^ diffused into the CSNWF and attached to the surface of the fibers. Afterward, the positive charged Ag^+^ formed complexes with hydroxyl groups. It is inferred that the hydroxyl groups anchor Ag^+^ and silver (Ag°) nucleation starts to take place after UV irradiation. UV irradiation can reduce Ag^+^ to Ag° [46]. It has proven to be an efficient, rapid and convenient approach for synthesizing AgNPs on various substrates [47–49]. In addition, the coordination between amine groups and Ag^+^ may decrease the potential of Ag+/Ag (E_Ag+/Ag_) and thus promote the reduction of Ag^+^ [50]. Consequently, the AgNPs were grown and formed based on these nuclei.

The size of the AgNPs was calculated in the range of 10–300, 90–380 and 100–500 nm when the concentration of Ag^+^ was 5, 10 and 20 mM, respectively (Fig. 1d). Meanwhile, the density of AgNPs was increased with the Ag^+^ increasing. However, micrometer scale and clustered particles were observed when the concentration of Ag^+^ was 50 mM (Supplementary Fig. S5). The results suggested that the available Ag^+^ concentration for AgNPs synthesis was 5–50 mM when the UV radiation time was 30 min. In addition, the effect of UV irradiation time on the AgNPs formation was investigated by exposing the AgNO_3_ impregnated CSNWF to UV at different times. Consequently, long-term UV irradiation could increase the amount and size of AgNPs (Fig. 1e). Monodisperse AgNPs (140.9 ± 59.2 nm) were obtained when the UV irradiation was 45 min. However, a significant variation in particle size distribution was observed when the UV irradiation time was extended to 60 min (Fig. 1d). All results indicated that the size distribution and amount of the AgNPs were affected by the concentration of Ag^+^, and the intensity and duration of UV irradiation. Notably, the AgNPs evenly distributed all surfaces of fibers without any aggregation or accumulation.

**Figure 2.**
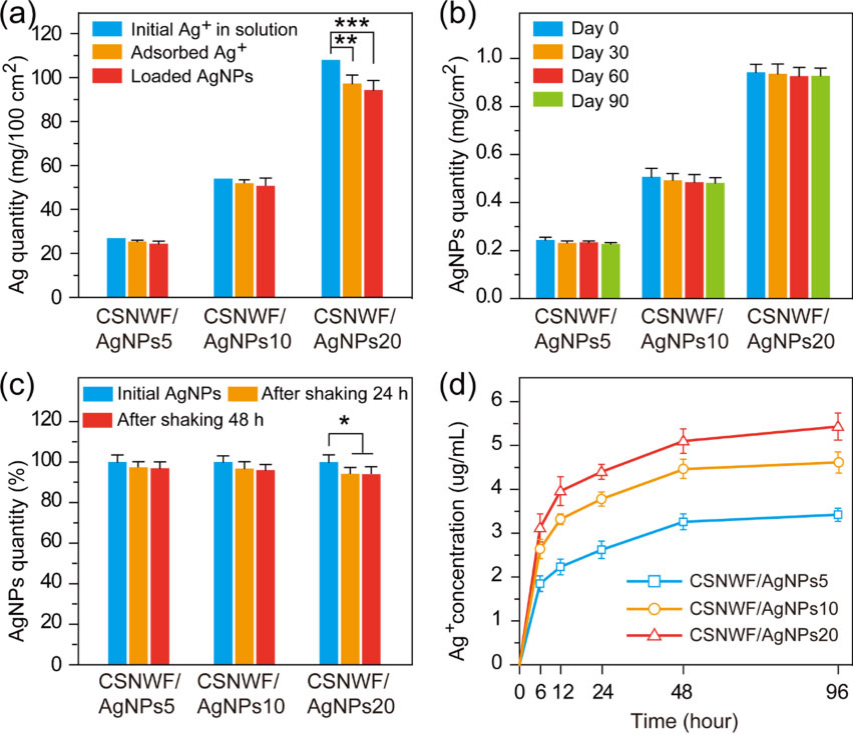
Physical and chemical properties of AgNPs on CSNWF. (**a**) Ag^+^ adsorption capacity and AgNPs loading efficiency on CSNWF. (**b**) The stability and (**c**) the adhesive ability of AgNPs on fibers of CSNWF. (**d**) Ag^+^ release curve of CSNWF/AgNPs in PBS (*P < 0.05, **P < 0.01 and ***P < 0.001)

The physical and chemical properties of AgNPs on CSNWF were investigated. The CSNWF showed excellent adsorption capacity for Ag^+^, and 94.19 ± 2.36% (CSNWF/AgNPs5), 96.23 ± 3.08% (CSNWF/AgNPs10) and 90.20 ± 3.98% (CSNWF/AgNPs20) of Ag^+^ were absorbed within 1 h (Fig. 2a). From the adsorption curves of Ag^+^ on CSNWF in Supplementary Fig. S6, we can found that ∼80% of Ag^+^ absorbed at the initial 10 min when the concentration of Ag^+^ ranging from 1 to 50 mM. The adsorption process became slow and reached saturated around 40 min. In addition, the amount of AgNPs loaded on each groups of the CSNWF was 90.37 ± 4.92% (CSNWF/AgNPs5), 93.89 ± 6.10% (CSNWF/AgNPs10) and 87.31 ± 4.56% (CSNWF/AgNPs20) compared with the initial amount of Ag^+^. The results indicated that the UV irradiation method is highly efficient, which can reduce 96–98% of adsorbed Ag^+^ into AgNPs.

The stability and adhesive ability of AgNPs on fibers of CSNWF were associated with the timeliness of antimicrobial and potential damage to the human body (toxicity of nanoparticles). No significant change in AgNPs amount was observed during 90 days of storing (Fig. 2b), which indicated that the high stability of AgNPs on CSNWF. Moreover, the adhesive ability of AgNPs on CSNWF was investigated by rude oscillation for two consecutive days. Consequently, no obvious loss of AgNPs in both groups of CSNWF/AgNPs5 and CSNWF/AgNPs10, while a slight amount of AgNPs (6.1%) was lost in CSNWF/AgNPs20 (Fig. 2c). Therefore, the strong adhesive ability of AgNPs on CSNWF can prevent potential damage to the body caused by nanoparticle detachment. The reason mainly attributes to the interaction of amine groups and hydroxyl groups on CS with Ag^+^, and CS acts as a stabilizer during the synthesis of AgNPs [51]. Furthermore, the AgNPs were expected to be used as a source of Ag^+^ and sustain the release of Ag^+^, thereby achieving a long-term antibacterial effect. The results of the Ag^+^ release test indicated that the Ag^+^ was released in a manner of sustain and slow (Fig. 2d). All results demonstrated that the presented AgNPs have excellent stability and adhesive ability on CSNWF, which has a long-term antibacterial effect as well as avoid the risk of the nanoparticles’ entry into the human body.

### High antimicrobial activity

**Figure 3.**
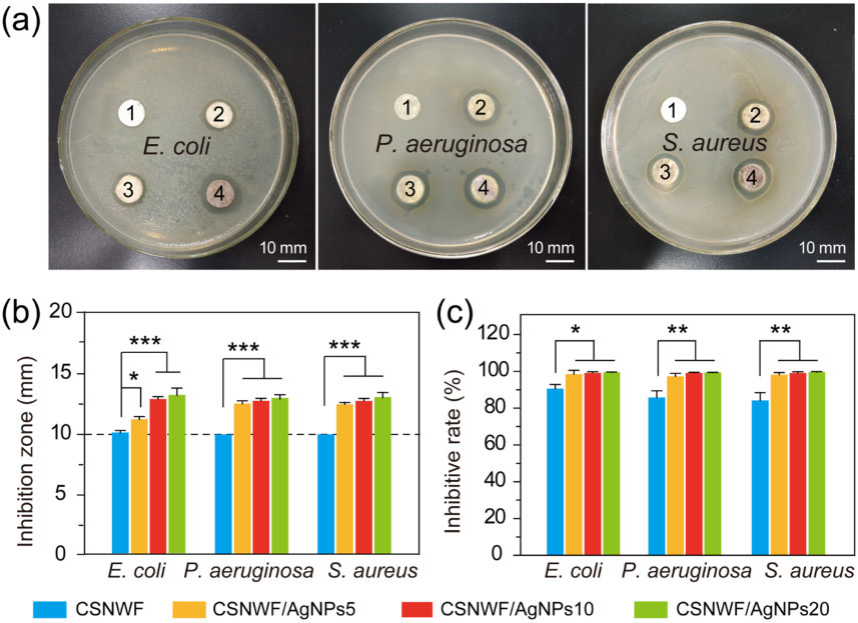
Antimicrobial properties of CSNWF/AgNPs and CSNWF. (**a**) Photographs of inhibition zones of (1) CSNWF, (2) CSNWF/AgNPs5, (3) CSNWF/AgNPs10 and (4) CSNWF/AgNPs20 against *E.coli*, *P.aeruginosa* and *S.aureus*, and the relative diameters of inhibition zones calculated in (**b**). (**c**) Bacterial inhibitive rate of CSNWF/AgNPs and CSNWF to *E.coli*, *P.aeruginosa* and *S.aureus* by shake flask method (*P < 0.05, **P < 0.01 and ***P < 0.001)

The antibacterial mechanism of AgNPs predominantly through oxidative damage caused by the generation of reactive oxygen species, as well as the strong interaction between released Ag^+^ and thiol groups present in the respiratory enzymes in the bacterial cell [52, 53]. The antimicrobial activity of CSNWF/AgNPs was evaluated by the inhibition zone method and shake flask method with pure CSNWF as a comparison. *Escherichiacoli*, *P.aeruginosa* and *S.aureus* were selected as representing bacteria that prevalent pathogenic bacterial strains in the wound site. Clear-cut inhibition zones were observed around all CSNWF/AgNPs groups for all bacteria (Fig. 3a). In contrast, the CSNWF group showed no obvious inhibition zones, even though it reported has intrinsic antimicrobial activity. The diameters of inhibition zones for each group were calculated and summarized in Fig. 3b. The results suggested that the antimicrobial efficacy of CSNWF/AgNPs with different amounts of AgNPs loading has a negligible difference between *P.aeruginosa* and *S.aureus*. The remarkable antimicrobial activity of CSNWF/AgNPs was further quantitatively confirmed by the shake flask method. The inhibition rate of CSNWF/AgNPs for *E.coli*, *P.aeruginosa* and *S.aureus* were detected above 98.55%, 97.36% and 98.21%, respectively, while the CSNWF was measured at 90.65 ± 2.32%, 85.80 ± 3.66% and 84.26 ± 4.25% accordingly (Fig. 3c). Comprehensive of the loading amount and size distribution of loaded AgNPs, and antibacterial activity, CSNWF/AgNPs10 was selected as an interlayer for composite wound dressing preparation.

### Physical and mechanical properties of composite wound dressings

**Figure 4.**
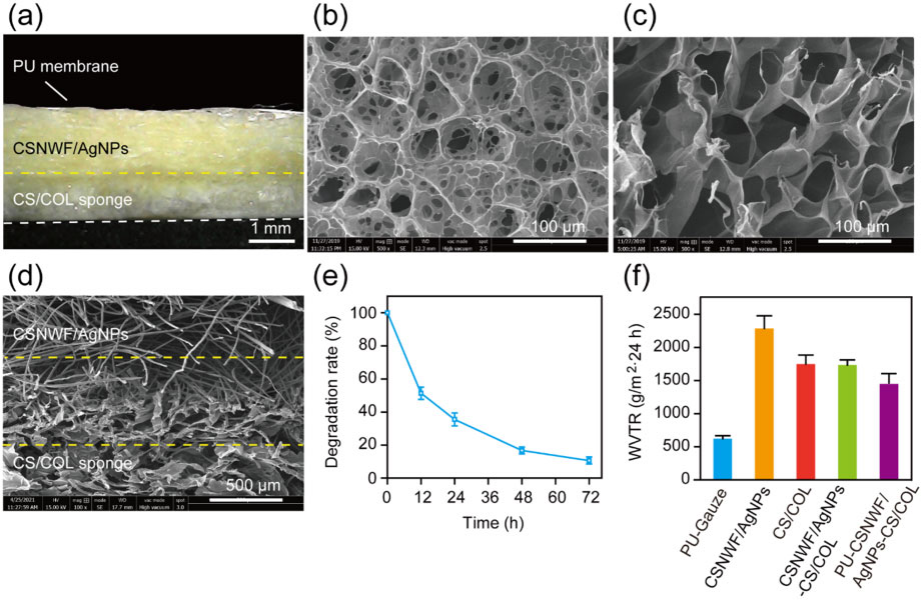
Physical and mechanical properties of the sandwich structure composite wound dressing. (**a**) Digital microscope image of the cross-section of the composite wound dressing. Scanning electron microscope (SEM) images of the (**b**) surface and (**c**) cross-section of the CS/COL sponge (500×). (**d**) SEM image of the interface between the layer of CSNWF/AgNPs and CS/COL sponge. (**e**) Biodegradation rate of the CS/COL sponge. (**f**) WVTR of the wound dressing

The sandwich structure composite wound dressing was fabricated by the outer layer of the PU membrane, the interlayer of CSNWF/AgNPs and the inner layer of the CS/COL sponge (Fig. 4a). The PU membrane with a nanoporous structure can maintain the exchange of water vapor and also acts as a protective barrier to prevent the invasion and contamination of external bacteria, dust, oil and water. In addition, the PU membrane with superior stretchability provides sufficient mechanical strength for the composite wound dressing, which is convenient for clinical application. The CS/COL sponge was obtained by spaying the homogeneous CS/COL mixture directly on the CSNWF/AgNPs and then lyophilized without crosslinking treatment. The prepared CS/COL sponge exhibits a homogeneous microporous network structure (Fig. 4b and c and Supplementary Fig. S7), and it was tightly bonded with the layer of CSNWF/AgNPs (Fig. 4d).

In our work, the CS/COL sponge inner layer was in direct contact with the wound bed. The soft sponge was comfortable for use and can reduce patient suffering. Furthermore, it was expected to quickly absorb exudates and maintenance of a moist milieu. The water absorption capacity of the CS/COL sponge was measured to be 298.25 ± 34.59%. The high water absorption capacity was mainly due to the high hydrophilicity of COL and porous network structure. In addition, the result of the CS/COL sponge biodegradation indicated that it could maintain ∼20% of weight within 2 days, even without any crosslinking treatment (Fig. 4e). It was mainly related to the adding CS component, which increases the hydrophobic of the sponge and thus declines the rate of the swelling process. Consequently, the CS/COL sponge has enhanced structural stability. The degradation period was sufficient for wound dressings application, which usually changed in 2 or 3 days. The breathability of the wound dressing was evaluated by a water vapor transmission rate (WVTR) experiment, and the value was 1447.26 ± 123.86 g/m^2^·24 h (Fig. 4f). In general, a higher WVTR leads to wound tissue dehydration and scar formation. In contrast, a lower WVTR resulted in the tissue’s abnormal metabolism, which could delay healing and increase the risk of bacterial infection. The developed wound dressing with a moderate WVTR value was able to maintain an optimal moisture content for the proliferation of epidermal cells and fibroblasts, thus suitable for wound healing applications.

### *In vitro* biocompatibility and wound healing evaluation

**Figure 5.**
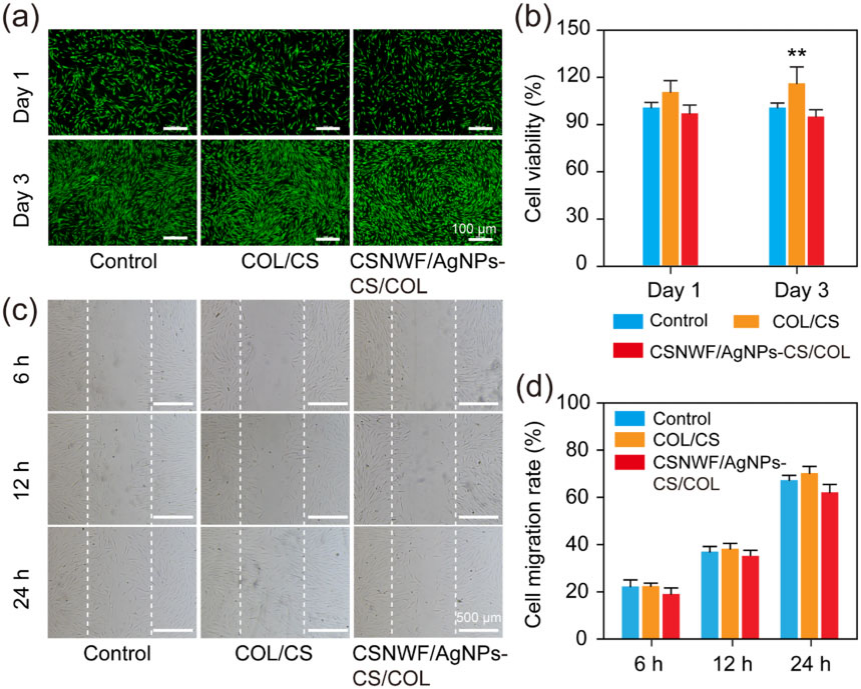
*In vitro* biocompatibility and wound healing evaluation of composite wound dressings. (**a**) Fluorescence images of HSF cells after live/dead staining. Green fluorescence presented living cells, and the red fluorescence indicates dead cells. (**b**) The proliferation of HSF cells versus different culture times by WST-1 assay. The optical density value of the control group on Day 1 was denoted as the viability of 100%. (**c**) Representative images of HSF cells migration into the ‘wound gap’. (**d**) Cell migration rates. The control group was the cells seeded in the normal cell culture medium (**P < 0.01 versus the control group)

The *in vitro* biocompatibility of the developed composite wound dressing was evaluated by culture the HSF cells in the leaching solution of CS/COL sponge and CSNWF/AgNPs-CS/COL. The viability of HSF cells was visualized by the live/dead staining. It was clear that most of the cells were alive in the leaching solution of CS/COL sponge and CSNWF/AgNPs-CS/COL, and no noticeable difference from control groups (Fig. 5a). In addition, WST-1 quantitative assay results further confirmed that the wound dressing group’s cell viability was comparable to control groups (Fig. 5b). It was worth noting that the cell density of CS/COL sponge group was slightly higher than the control group, which might be attributed to the nutritional function of COL composition. The results indicated that the cytotoxic of the wound dressing prepared by CS/COL sponge and CSNWF/AgNPs can be ignored.

The *in vitro* wound healing effect was evaluated by investigation of the migratory activities of HSF cells in leaching solutions. HSF cells in the leaching solution migrated into the wound area (blank area) same as the cells in the normal culture medium (Fig. 5c). By 24 h, HSF cells in the leaching solution of CS/COL and CSNWF/AgNPs-CS/COL had covered 70.28 ± 2.17% and 62.12 ± 2.69% wound area, respectively, while HSF cells in the control group had covered 67.32 ± 1.35% wound area (Fig. 5d). The enhanced migration rate in CS/COL group was mainly due to the composition of CS and COL, which were favorable for HSF migration. However, the slightly reduced cell migration rate in CSNWF/AgNPs-CS/COL group may be due to the released Ag^+^.

### *In vivo* evaluation of wound healing effect

**Figure 6.**
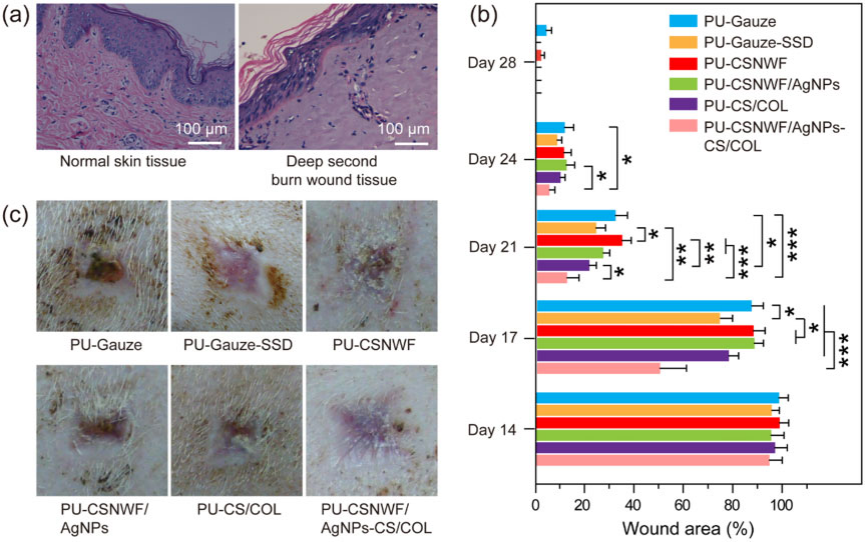
(**a**) Histological observation of porcine skin tissue structure on normal and the deep second burn wound area by H&E staining. (**b**) Percent change in wound area over a 4-week period. (**c**) Photographs of the wound healing effect in all groups on Day 28 post-surgery (*P < 0.05, **P < 0.01 and ***P < 0.001)

The effect of AgNPs anchored composite wound dressing for severe burn wound healing was evaluated in a porcine model. A deep second burn wound was created by the thermal scalding approach and confirmed by H&E staining. As shown in Fig. 6a, the epithelial cells have well-defined morphology and were arranged neatly, continuously, with clear layers in healthy skin tissue. After the scald, the epithelial cells partially fell off from the wound site, and some cells dissolved. Degeneration and necrosis were observed in the wound site, and its structure became blurred. The interstitial tissue of the dermis was loose and congested, and the COL fibers fused into pieces. The entire epidermis has been destroyed, and partial dermal tissue was damaged after the scald, which indicated that the deep second-degree burn model was successfully established.

To eliminate the interference of body parts and individual differentiation, the created burn wounds were randomly administered, and eight pigs (including 160 wound beds) were used in our work. The wound healing effect of the presented wound dressing PU-CSNWF/AgNPs-CS/COL was compared with various control groups, including PU-gauze, PU-gauze-SSD, PU-CSNWF, PU-CSNWF/AgNPs and PU-CS/COL sponge. Herein, commercial available antibacterial agent SSD is widely used in wound treatment as a positive control group, whereas the medical cotton gauze as the negative control group. A 4-week period of the wound healing process was recorded, and the wound closure was investigated at first.

No significant changes in wound area were observed during the initial 2 weeks, and the wound closure in all groups was below 5% (Fig. 6b). Compared with the PU-gauze group, the PU-CSNWF/AgNPs-CS/COL, PU-CS/COL and PU-SSD have a slightly higher healing process at Day 14 post-surgery. Moreover, the tendency became more evident on Day 17, in which the wound area of PU-CSNWF/AgNPs-CS/COL, PU-CS/COL and PU-SSD was calculated as 49.8 ± 9.85%, 77.6 ± 3.88% and 73.9 ± 4.05%, respectively, whereas other groups remain over 85%. The accelerated wound healing process was observed in all groups from the third week, while the PU-CSNWF/AgNPs-CS/COL maintained a leading edge, followed by the PU-SSD and PU-CS/COL sponge. Meanwhile, rapid scab formation and fewer wound exudate amounts were observed in PU-CSNWF/AgNPs-CS/COL (Supplementary Fig. S8). On Day 28, most groups were wholly closed except for the PU-gauze and PU-CSNWF. In addition, the represented healed wound in all groups on Day 28 were exhibited in Fig. 6c. A smooth and flat regenerated skin occurred at the wound bed in PU-CSNWF/AgNPs-CS/COL. In contrast, an irregular regenerated skin was observed in the positive control group PU-gauze-SSD, PU-CSNWF/AgNPs and PU-CS-COL, even though they have completely closed. Moreover, the wound on groups of PU-gauze and PU-CSNWF remains unclosed, and a few crusts exist in these groups. The results indicated that the healing effect of developed composite wound dressing was superior to other groups.

**Figure 7.**
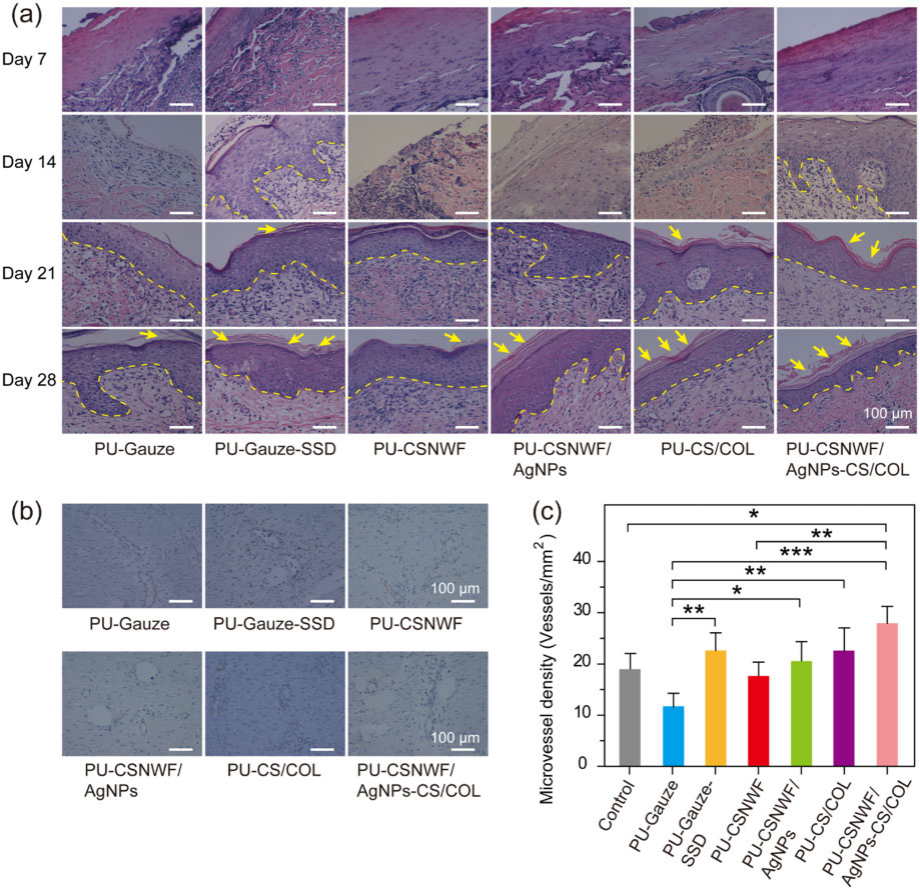
Reepithelialization and angiogenesis in wound sites. (**a**) Representative images of histological sections with H&E staining on Day 7, Day 14, Day 21 and Day 28 post-surgery. The yellow dashed line indicates the boundary between the newly formed epidermis and dermis. The yellow arrow represents the stratum corneum. (**b**) Representative images of immunohistochemical staining of wound sections at Day 28 with factor VIII. (**c**) Quantification of vessel number per microscopic field in different groups (*P < 0.05, **P < 0.01 and ***P < 0.001)

The wound healing process was further evaluated by H&E staining of the tissue section on wound sites. A necrotic epidermal layer observed in all groups on Day 7, and the pore structures under the epidermis indicated blisters occurred after scalding (Fig. 7a and Supplementary Fig. S9). A clear newly formed squamous epithelial layer and fewer lymphocyte infiltration were found in PU-CSNWF/AgNPs-CS/COL and PU-Gauze-SSD on Day 14, whereas other groups showed no epithelial layer. In particular, the severe inflammatory response was found in groups of PU-Gauze and PU-CSNWF. Regenerated epidermal was observed in all groups at Day 21, and no obvious evidence of lymphocyte infiltration in PU-CSNWF/AgNPs-CS/COL. Clear stratum corneum, epidermis and dermis structures were observed in groups of PU-Gauze-SSD, PU-CSNWF/AgNPs, PU-CS/COL sponge and PU-CSNWF/AgNPs-CS/COL at Day 28, indicated that they completely rehabilitated. However, just a thin layer of stratum corneum showed in PU-Gauze and PU-CSNWF, which consistent with the wound closure results. The advanced reepithelialization and tight junction between the epidermis and dermis are essential for tissue functional and esthetic recovery. The results confirmed that prepared wound dressings could promote reepithelialization and new tissue formation, especially at the initial stage. The reason was mainly due to the released Ag^+^ inhibiting bacterial infections, thereby reducing the inflammatory response. Moreover, the CS and COL, especially the low molecule weight compositions, in composite wound dressings could stimulate the migration of fibroblasts and promote fibroblasts proliferation, respectively.

Angiogenesis plays a vital role in the wound healing process, which carries oxygen and nutrients to the wound. Tissue ischemia and hypoxia generally occur after scalding due to microvascular thrombosis, which can lead to wound aggravation and necrosis. Neovascularization provides adequate blood flow and oxygen for tissue repair, thereby promoting wound healing. No significant angiogenesis was observed during the first week (Supplementary Fig. S10). The neovascularization was remarkably increased in groups of PU-Gauze-SSD, PU-CS/COL and PU-CSNWF/AgNPs-CS/COL on Day 28 with the healthy tissue as a comparison (Fig. 7b and Supplementary Fig. S10). The density of blood vessel density in PU-CSNWF/AgNPs-CS/COL was measured as 28.0 ± 3.3/mm^2^. It was significantly higher than the PU-Gauze (11.7 ± 4.2/mm^2^) and slightly superior to the PU-Gauze-SSD (22.5 ± 5.0/mm^2^) (Fig. 7c). The results indicated that angiogenesis in the wound site was significantly improved by the PU-CSNWF/AgNPs-CS/COL, which could be attributed to the sustained Ag^+^ release can against infection and the synergistic effects of CS and COL.

### Variation of VEGF, NO and endothelin at the wound site

**Figure 8.**
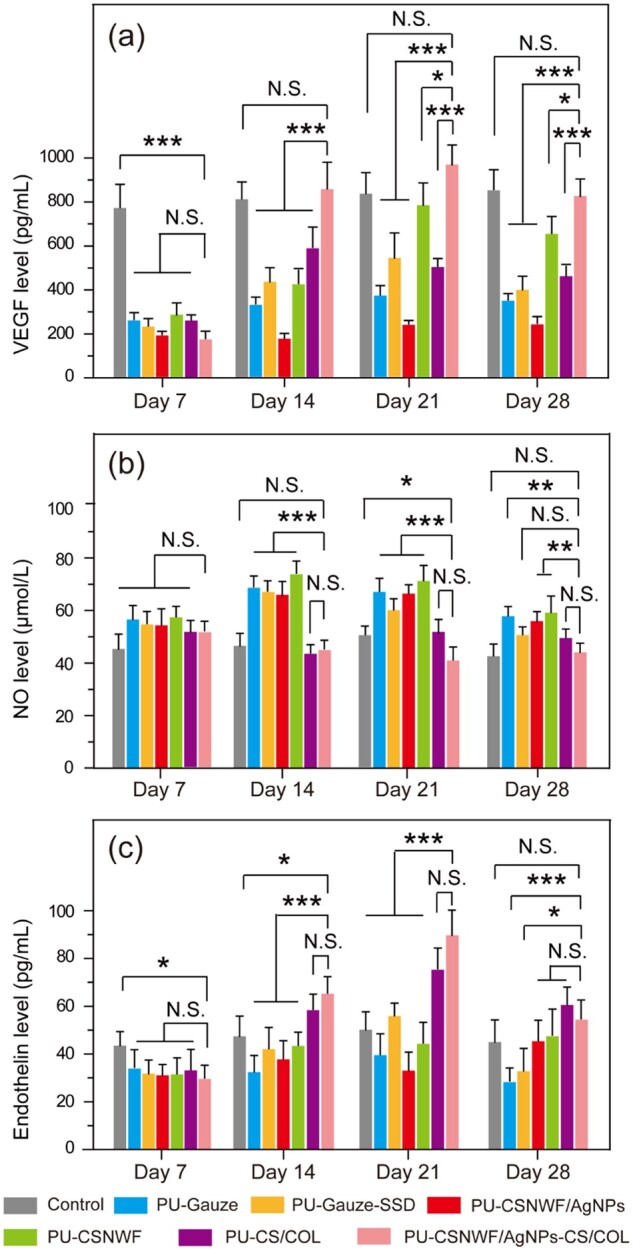
Variation of (**a**) VEGF, (**b**) NO and (**c**) endothelin level at wound sites during the wound healing process (N.S., no significant, *P < 0.05, **P < 0.01 and ***P < 0.001)

VEGF can promote angiogenesis by inducing and stimulating the chemotaxis and proliferation of endothelial cells [54]. Therefore, long-term and high levels of VEGF expression can help wound repair and tissue remodeling. In the first week after scald, all groups’ VEGF levels at the wound site were significantly lower than in the healthy group (Fig. 8a), and the results were consistent with the previous angiogenesis results. Afterward, a high level of VEGF was observed in the PU-CSNWF/AgNPs-CS/COL in the second and third weeks, and the value fell back on Day 28. In contrast, the PU-Gauze and PU-CSNWF were maintained at a low level during all periods. Although the VEGF level of PU-Gauze-SSD, PU-CSNWF/AgNPs and PU-CS/COL improved from the second week, the duration was shorter, and the content was lower than that of the PU-CSNWF/AgNPs-CS/COL. The results indicated that the PU-CSNWF/AgNPs-CS/COL could effectively stimulate the synthesis and release of VEGF.

NO and endothelin are playing an essential role in the process of wound deterioration and necrosis after burn injury. NO is a vasodilator substance *in vivo*. The content of NO is low in the physiological state, and it increases when tissue damage occurs [55]. High concentrations of NO could lead to inflammatory response aggravated in the early stage of wound healing [56]. Moreover, a large amount of NO in the wound site could promote the excessive expansion of the blood vessels and exudate secretion, causing tissue ischemia and hypoxia. And then leading to wound further deterioration and necrosis. In contrast, endothelin is a potent and long-lasting vasoconstrictor [57]. Normally, the synthesis and release of NO and endothelin are in dynamic equilibrium and regulate the diastolic state of the microvasculature.

The results showed that the content of NO in the wound site was higher than in the healthy tissues on Day 7 (Fig. 8b). Subsequently, the content of NO in groups of PU-Gauze, PU-Gauze-SSD, PU-CSNWF and PU-CSNWF/AgNPs was maintained at a high level during the whole healing period, whereas no significant difference was observed in the groups of PU-CSNWF/AgNPs-CS/COL and PU-CS/COL. Meanwhile, the endothelin content in all wound groups was significantly lower than that in the healthy group on Day 7 (Fig. 8c). Afterward, the groups of PU-CSNWF/AgNPs-CS/COL and PU-CS/COL grew faster, staying above the healthy group from the second week and peaking in the third week. It is worth noting that the NO content in all Ag-containing-group was higher than that of other groups, indicating that Ag cannot effectively inhibit the production of NO. However, the PU-CSNWF/AgNPs-CS/COL can promote the high-efficiency expression of VEGF, induce the formation of blood capillaries and stimulate its division and proliferation, and thus promote wound repair and tissue reconstruction.

## Conclusions

A sandwich structure composite wound dressing with high biocompatibility and antimicrobial activity was developed for severe burn wound healing. The antibacterial agent AgNPs were *in situ* synthesized on the fiber of CSNWF. The strong adhesion between AgNPs and CS could eliminate AgNPs aggregation and shedding, and thus maintain high antimicrobial activity and minimal toxicity. Meanwhile, the sustained released Ag^+^ could inhibit bacterial infections, thereby reducing the inflammatory response of the wound site. Moreover, the composition of CS and COL in composite wound dressings can accelerate the wound healing process. The *in vivo* wound healing effect of the presented wound dressing on deep dermal burn was demonstrated in a porcine model. The results indicated that the prepared wound dressing has excellent reepithelialization and angiogenesis properties through the promotion of the VEGF and endothelin expression as well as inhibition of NO production. Therefore, our finding suggested that the prepared AgNPs anchored sandwich structure composite wound dressing can be represented as a promising antimicrobial wound dressing for severe wound healing.

## Supplementary data

Supplementary data are available at *REGBIO* online.

## Funding

This work was supported by the National Natural Science Foundation of China (31800796), the Nature Sciences Funding of Fujian Province (2019J01238) and the Fuzhou University Testing Fund of Precious Apparatus (2021T018).

*Conflict of interest statement*. None declared.

## Supplementary Material

rbab037_Supplementary_Data
