# Supplementary material for: A sandwich structure composite wound dressing with firmly anchored silver nanoparticles for severe burn wound healing in a porcine model
Source: Regen Biomater. 2021 Aug 3;8(5):rbab037. doi: 10.1093/rb/rbab037 (PMC8329475; doi:10.1093/rb/rbab037)
Supplement: rbab037_Supplementary_Data [file rbab037_supplementary_data.docx]

**Supporting Information**

**A sandwich structure composite wound dressing with firmly anchored silver nanoparticles for** **severe burn wound healing** **in a porcine model**

Jianmin Yang ^1,2^, Yufeng Huang ^1^, Jiajia Dai ^1^, Xianai Shi ^1,2^, Yunquan Zheng ^2,3,*^

^1^ College of Biological Science and Engineering, Fuzhou University, No. 2 Xueyuan Road, Fuzhou, 350108, China

^2^ Fujian Key Lab of Medical Instrument and Biopharmaceutical Technology, Fuzhou University, No. 2 Xueyuan Road, Fuzhou, 350108, China

^3^ College of Chemistry, Fuzhou University, No. 2 Xueyuan Road, Fuzhou, 350108, China

* Correspondence address. College of Chemistry, Fuzhou University, No. 2 Xueyuan Road, Fuzhou, 350108, China. E-mail: yunquanzheng@fzu.edu.cn.

**1. Characterization**

**SEM&EDS characterization.** The micromorphology of CSNWF/AgNPs, CS/COL sponge, and the composite wound dressings was observed by a Hitachi S4800 SEM (Tokyo, Japan). All specimens were sputter-coating with gold before SEM observation. During SEM tests, EDS (INCA X-Max 250) spectra were collected to analyze the chemical elements of the samples.

**Size distribution of AgNPs.** At least 300 particles in each sample (SEM images) were measured using the ImageJ software. The monodispersity of particles was evaluated by measuring the mean particle size and the diameter coefficient of variation (CV). The size distribution of particles was represented by a fitted curve.

**Adsorption capacity of CSNWF for Ag^+^.** The pretreated CSNWF with a size of 10 cm × 10 cm was immersed in 50 mL of AgNO_3_ aqueous solution (range from 1 mM to 50 mM). At predetermined time points, the Ag^+^ content of the solution was detected by ICP-OES (Optima 7000, PerkinElmer, USA). The Ag^+^ adsorption capacity (Q) was calculated as follows:

Q = V(C_0_－C_e_)/A (1)

In this equation, “V” is the volume of adsorbent solution, “C_0_” and “C_e_” are the initial and detected concentration of Ag^+^ in adsorbent solution, respectively, “A” is the area of CSNWF.

**AgNPs loading quantity test.** AgNPs/CSNWF with a size of 5 cm × 5 cm were soaked in 25 mL of 25% ammonia water for 2 h and then taken the supernatant. After repeating 3 times, the supernatants were mixed and the content of Ag^+^ was detected by ICP-OES.

**Investigation of the stability of AgNPs on CSNWF.** AgNPs/CSNWF with a size of 5 cm × 5 cm were individually packaged in a paper bag and then placed in an incubator at 45 °C with 70% humidity. At predetermined time points, the silver content on CSNWF was detected by ICP-OES.

**Evaluation of the adhesive ability of AgNPs on CSNWF.** AgNPs/CSNWF with a size of 2 cm × 2 cm were immersed in 100 mL of water and then placed in a shaker under 25 ^o^C. After 24 hours and 48 hours of constant shaking at 200 rpm, the AgNPs remaining on the CSNWF was detected by ICP-OES.

**X-ray diffraction (XRD) analysis.** The phase composition of the nanocomposites was analyzed by XRD, using a continuous mode from 10° to 80° of 2θ at a scanning speed of 5°/min on an X-Pert Pro (PANalytical) diffractometer equipped with a Cu Kα radiation source (λ = 0.15432 nm), operated at 40 kV and 30 mA.

**Sustainable release of Ag^+^ from the CSNWF/AgNPs.** CSNWF/AgNPs with size of 5.0 cm × 5.0 cm was immersed in 50 mL PBS (10 mM, PH = 7.4) and placed in a dark environment. At predetermined time points, 1 mL of the solution was collected, and then an equal amount of freshly prepared PBS was added. The collected solution was centrifuged at 8000 rpm for 10 min. Afterward, 0.5 mL of the supernatant was taken to mix with 0.5 mL of 1 M HNO_3_. After diluted with water, the concentration of Ag^+^ in the solution was directly determined by ICP-OES.

**2.** **Water** **absorption capacity and degradation of CS/COL sponge**

The CS/COL sponge was prepared by spaying the CS/COL mixture on a Teflon plate (10 cm in length × 10 cm in width × 2 cm in height), and the processes of degassing and lyophilizing were the same as wound dressing preparation. The prepared CS/COL sponge was cut into dimensions of 2.5 cm × 2.5 cm for the following test.

**Water absorption capacity test.** The CS/COL sponge samples were immersed in 50 mL PBS for 30 min and then removed the surface excess PBS by filter paper. The wet CS/COL sponge samples were weighted immediately, and the water absorption capacity (W) was calculated as follows:

W = (We－W_0_)/W_0_×100% (2)

In this equation, “W_0_” and “W_e_” are the weight of the CS/COL sponge before and after immersion in PBS.

**In vitro degradation test.** The CS/COL sponge samples were immersed in 50 mL PBS at 37 ^o^C with constant shaking (50 rpm). The weights of degraded samples were measured every day after water washing and lyophilization. The degradation percentage (D) of CS-COL sponges was calculated with the following formula:

D = (W_0_－W_d_)/W_0_ × 100% (3)

In this equation, where “W_0_” is the initial weight of the sample and “W_d_” is the weight after degradation.

**3.** **Water vapor transmission rate (WVTR) test**

The tested sample was cut into an appropriate disc and then sealed on the mouth of a cylindrical vial containing water. Afterward, the vial was placed into the incubator (HCJ-6D, Changzhou, China) with a temperature of 37 ^o^C and humidity of 75%. WVTR of samples was calculated as follows:

WVTR = (∆m/∆t)/A (4)

In this equation, “∆m/∆t” is the weight of moisture loss for 24 h, and “A” is the effective transfer area.


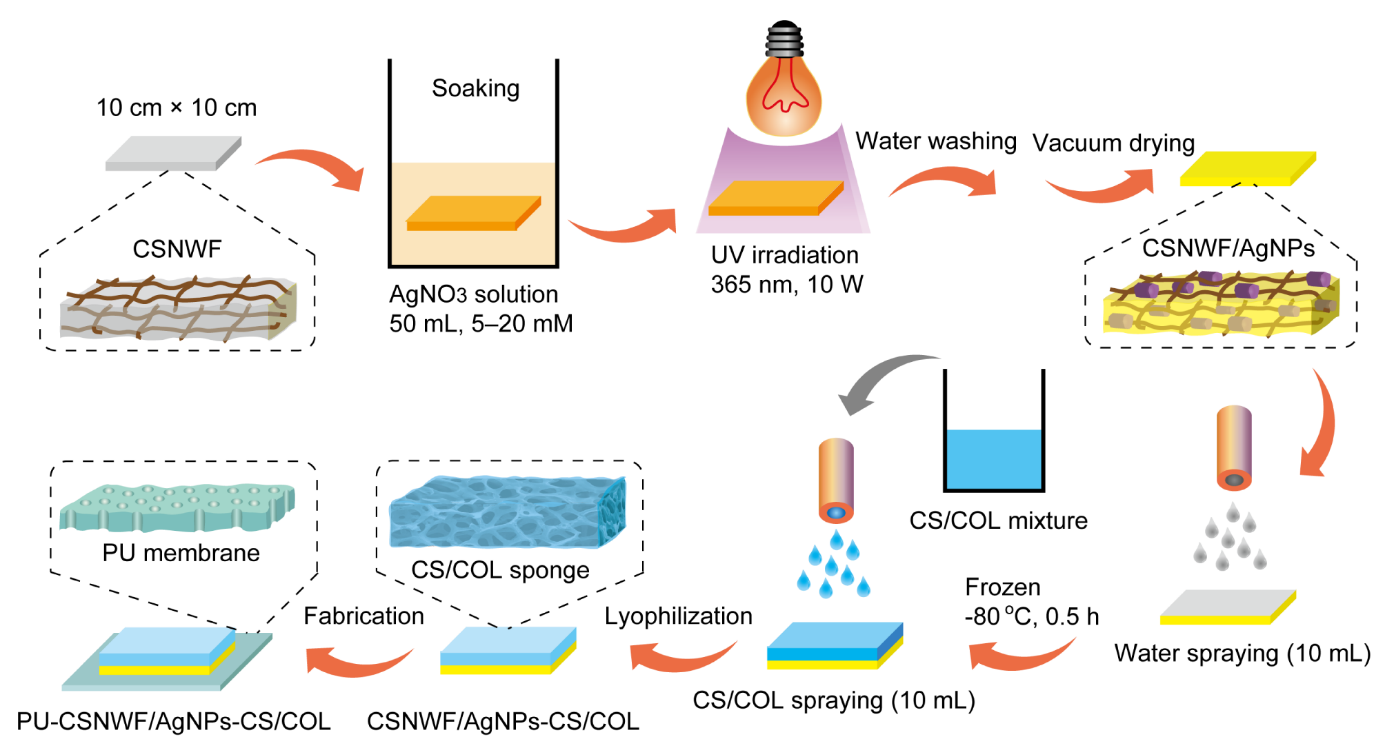


**Figure S1.** Schematic diagram of procedures for composite wound dressing preparation.


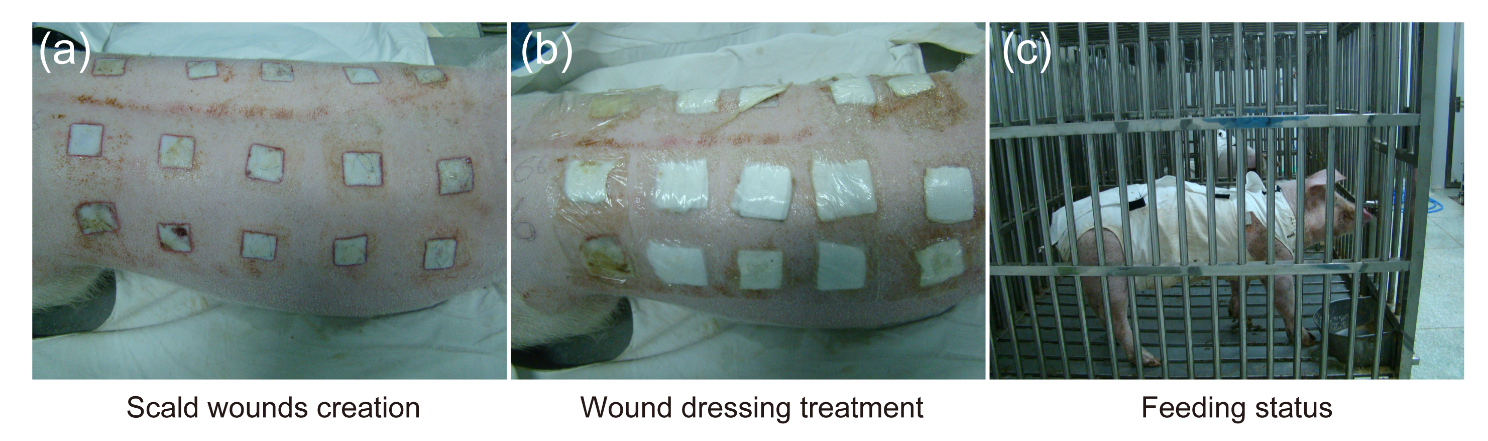


**Figure S2.** (a) Photograph of deep second burn wounds located on the dorsal of the pig (No. 6) after thermal scalding. (b) Wound beds covered with various wound dressings. (c) Pigs were fitted with custom-made garments and independent feeding.


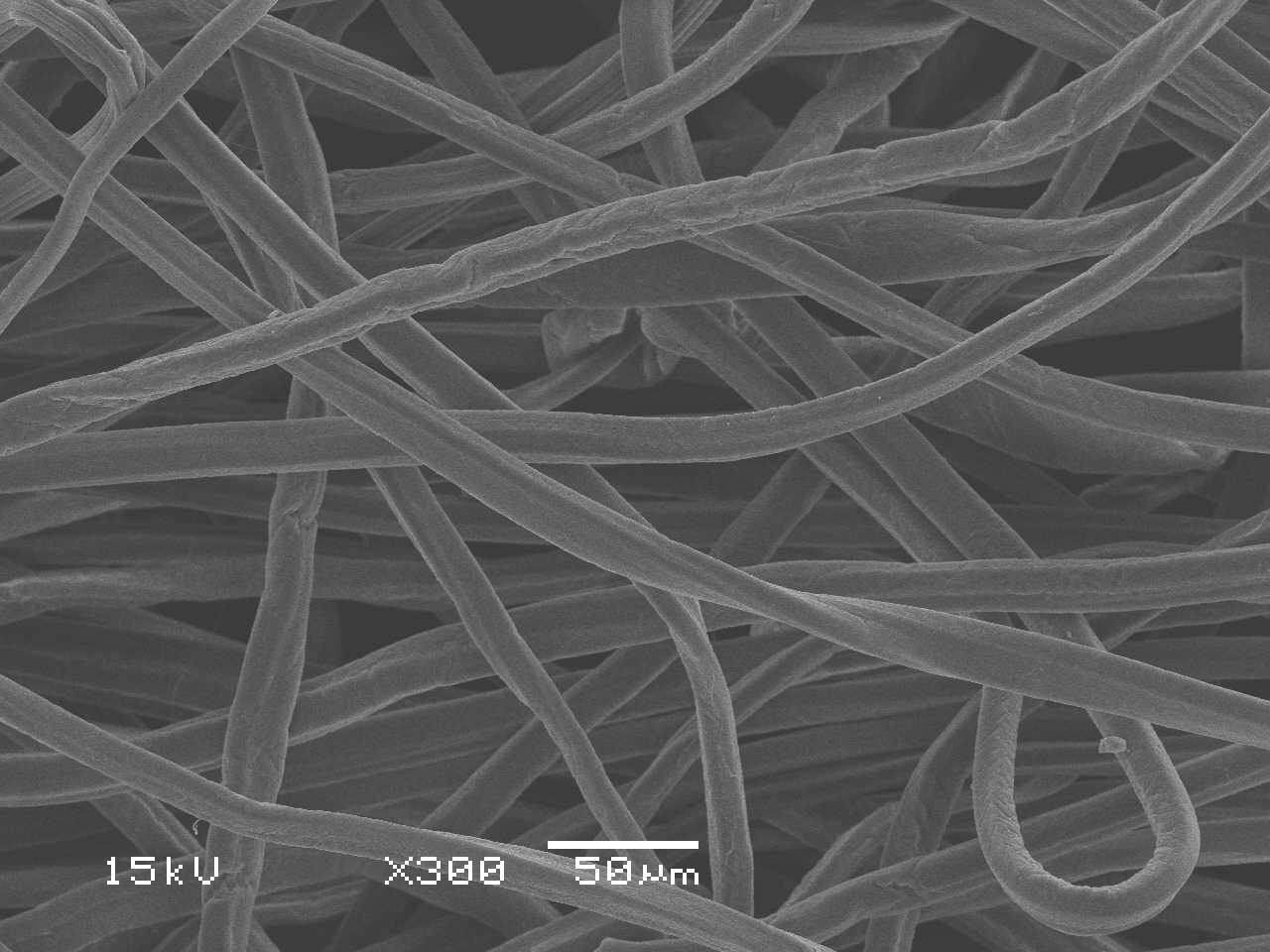


**Figure S3.** SEM images of chitosan nonwoven fabric (CSNWF).

**Figure S4** SEM images of AgNPs on the fiber surface of CSNWF without HNO_3_ pretreatment. The AgNO_3_ concentration was 20 mM.


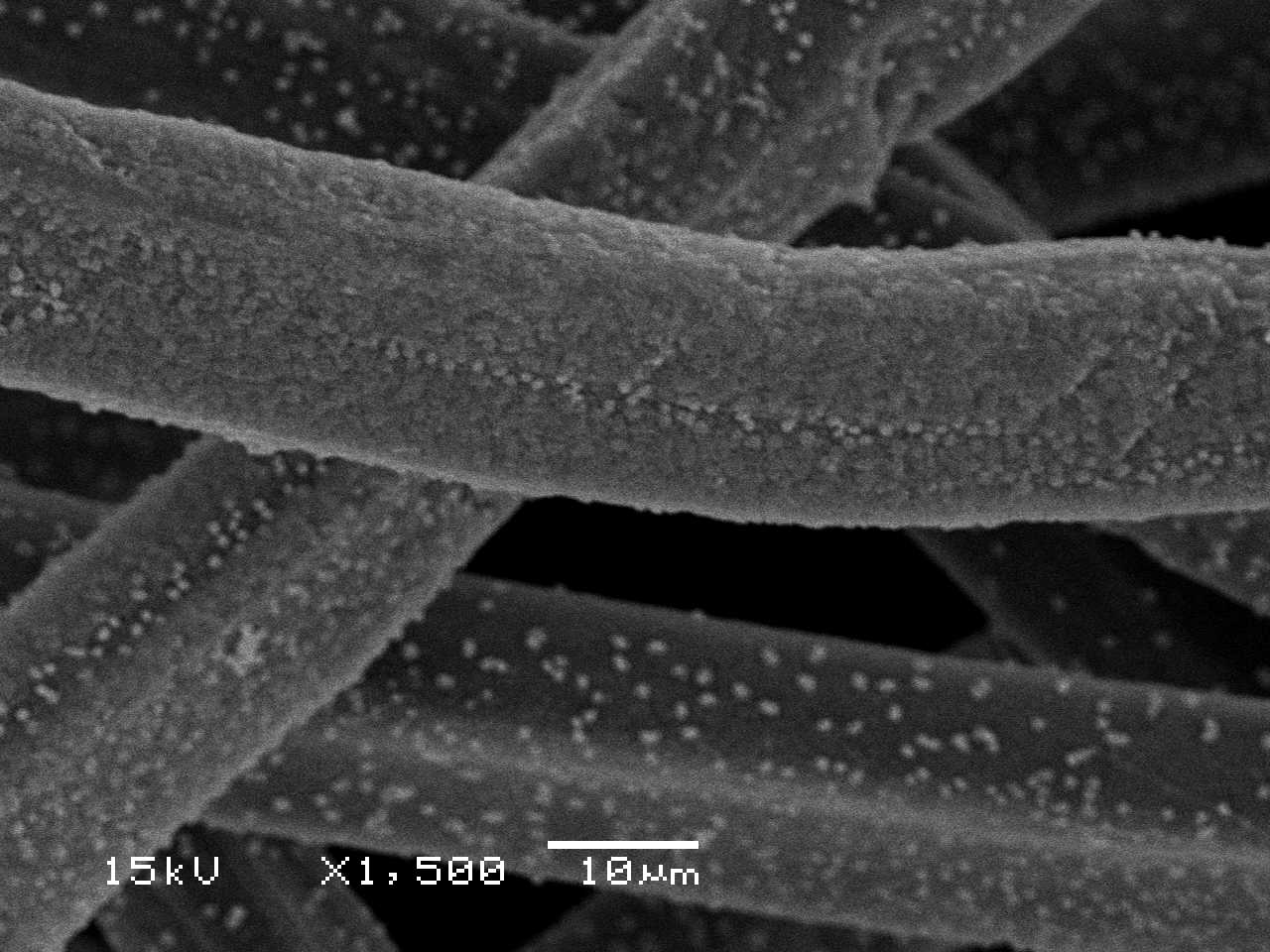


**Figure S5** SEM images of CSNWF/AgNPs50. The CSNWF was treated by the AgNO_3_ solution at the concentration of 50 mM, the UV irradiation time was 30 min.


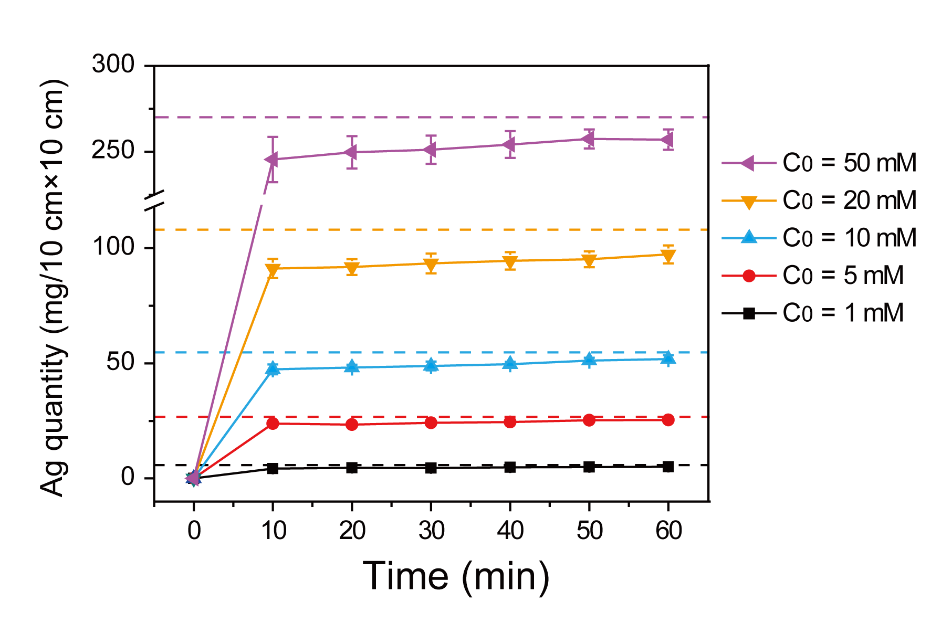


**Figure S6** Adsorption curves of Ag^+^ on CSNWF with different concentrations of AgNO_3_ solution. Dotted lines represent the total amount of Ag^+^ in the solution.

**
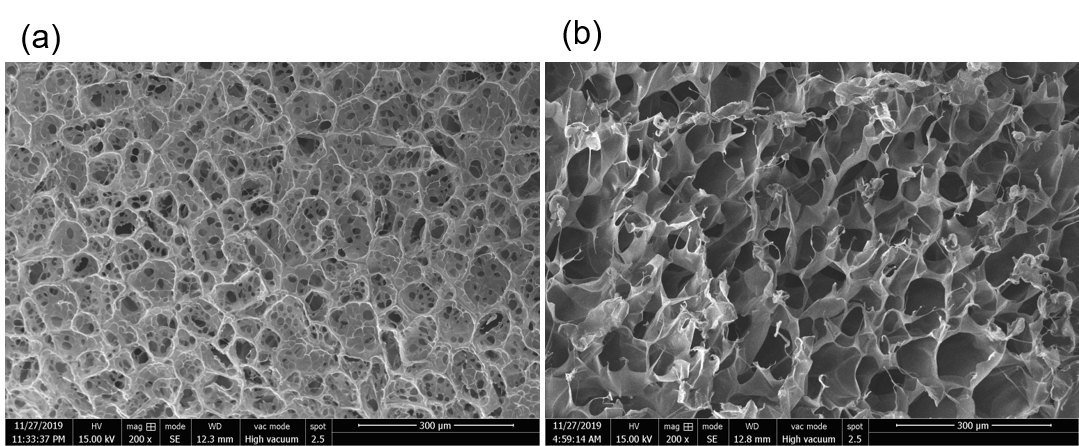
**

**Figure S7** SEM images of the (a) surface and (b) cross-section morphology of CS/COL sponge. (200 ×)

**
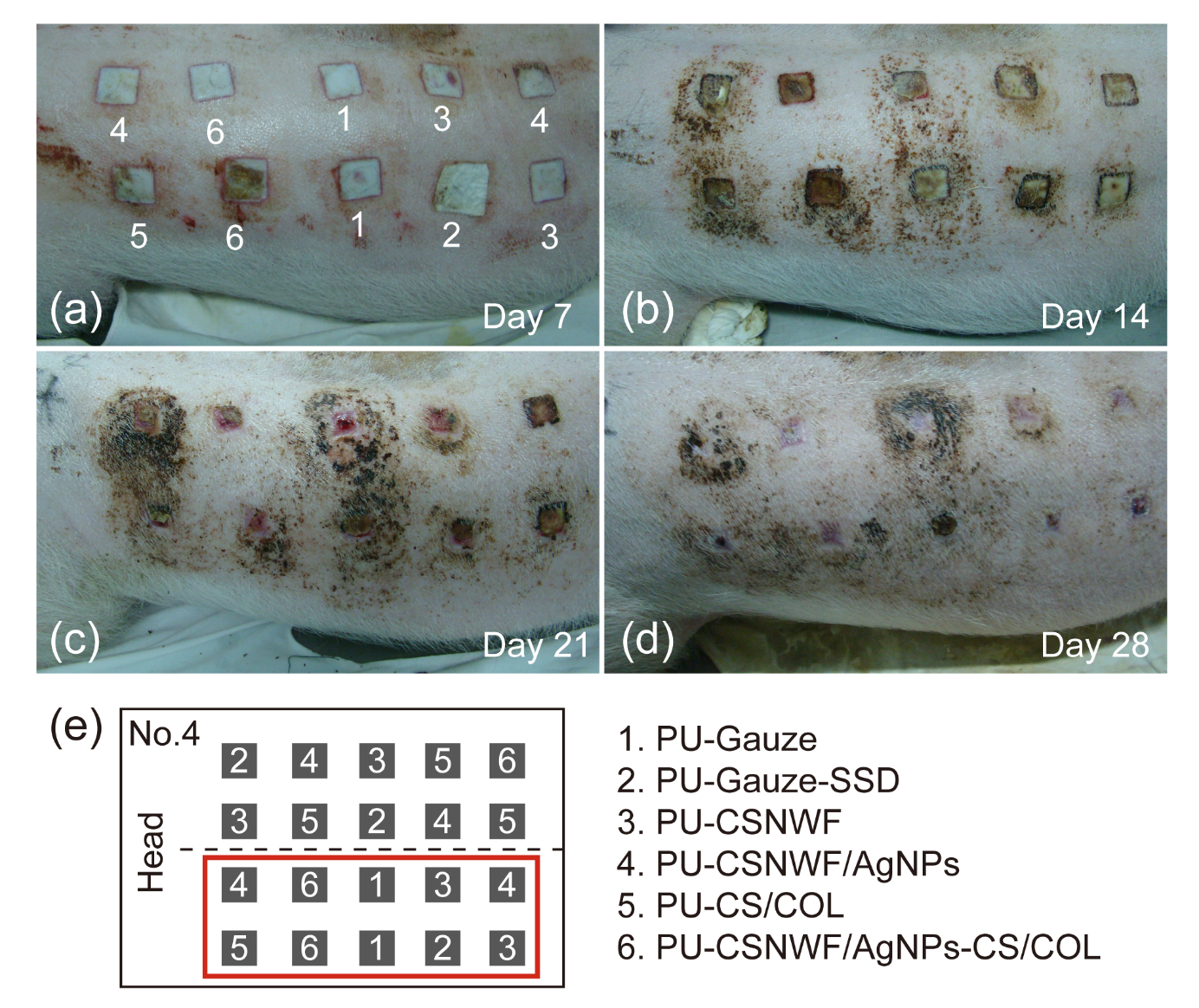
**

**Figure S8** (a-d) Photographs of the wound healing process on No. 4 pig at (a) day 7, (b) day 14, (c) day 21, and (d) day 28. (e) The numbering sequence of wound bed on No. 4 pig and the corresponding applied wound dressings.


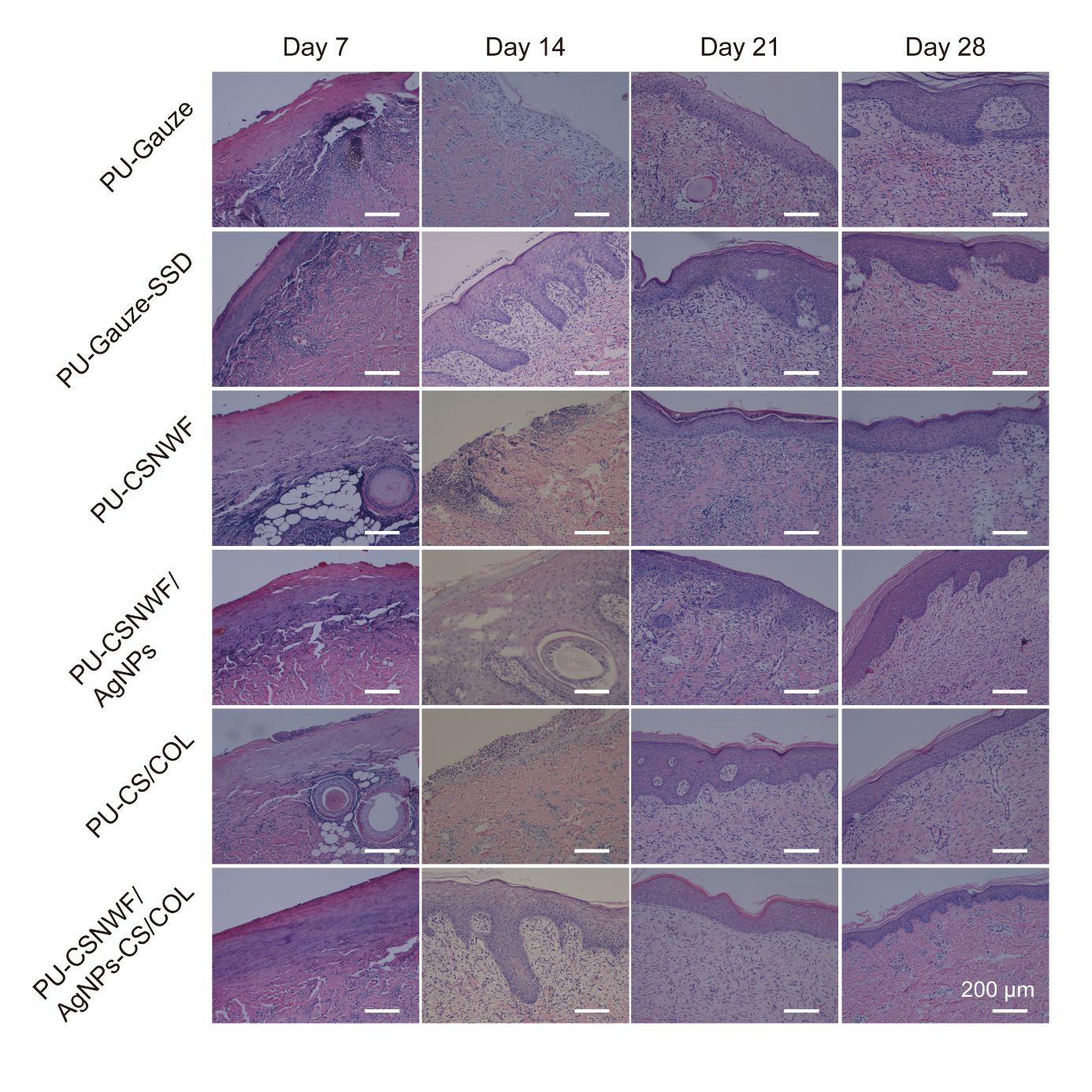


**Figure S9** Representative images of histological sections in groups of PU-Gauze, PU-Gauze-SSD, PU-CSNWF, PU-CSNWF/AgNPs, PU-CS/COL sponge, and PU-CSNWF/AgNPs-CS/COL with H&E staining at day 7, day 14, day 21, and day 28. All images were the enlarged field of view based on **Fig. 7a**.


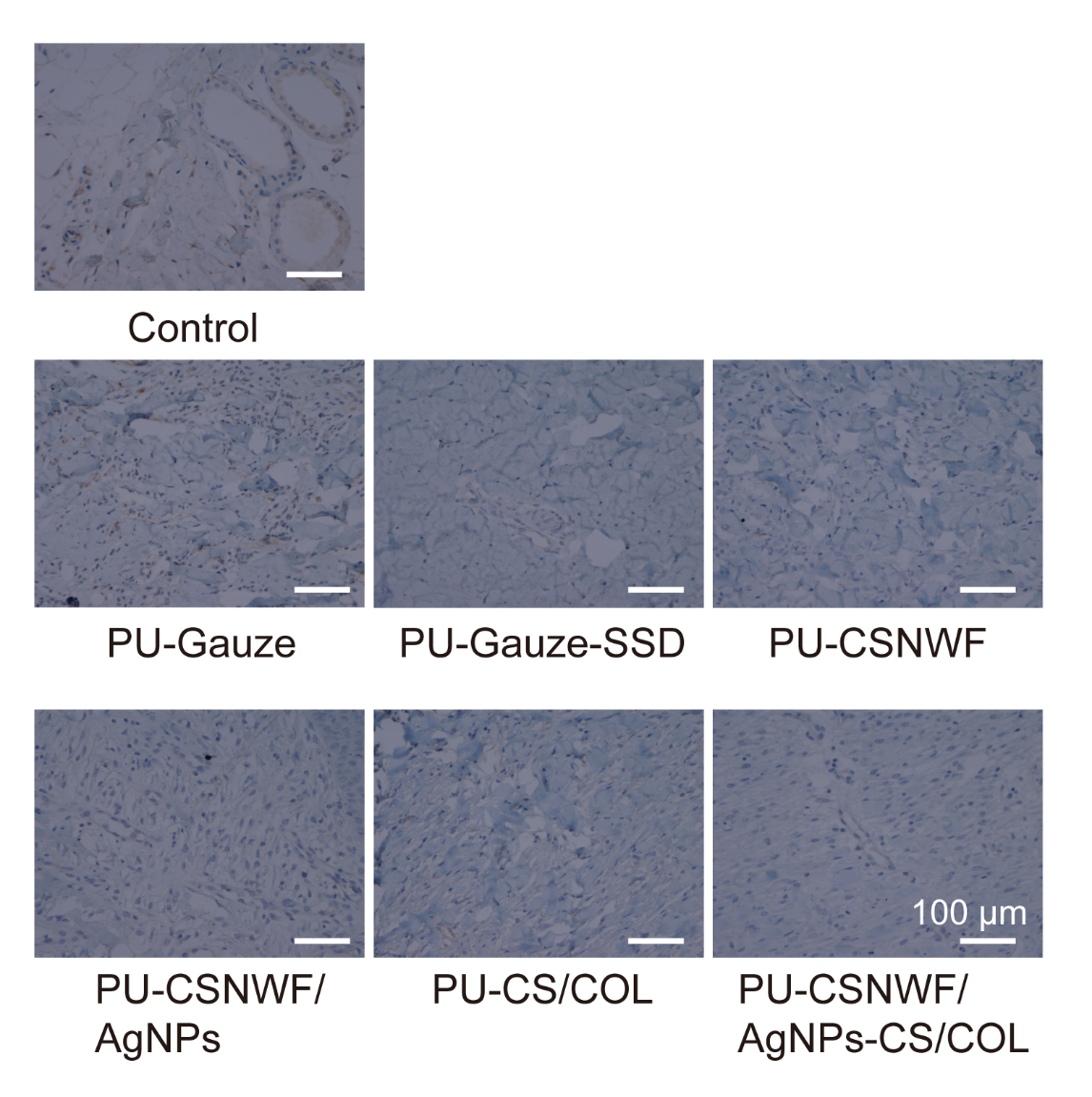


**Figure S10** Representative images of immunohistochemical staining of wound sections at day 7 with factor VIII. The control group means normal tissue site.
